# Silk-ELR co-recombinamer covered stents obtained by electrospinning

**DOI:** 10.1093/rb/rby022

**Published:** 2018-10-30

**Authors:** M Putzu, F Causa, Manuel Parente, Israel González de Torre, J C Rodriguez-Cabello, P A Netti

**Affiliations:** 1 Dipartimento di Ingegneria Chimica, dei Materiali e della Produzione Industriale (DICMAPI), University “Federico II”, Piazzale Tecchio 80, Naples, Italy; 2 Interdisciplinary Research Centre on Biomaterials (CRIB), University of Naples Federico II, Piazzale Tecchio 80, Napoli, Italy; 3 Center for Advanced Biomaterials for Health Care@CRIB, Istituto Italiano di Tecnologia, Largo Barsanti e Matteucci 53, Napoli, Italy; 4 Conic Vascular, via Carlo Maderno 23, Lugano, Switzerland; 5 Technical Proteins NanoBioTechnology S.L., Edificio CTTA, Paseo de Belen, 9A, Valladolid, Spain; 6 Networking Research Center on Bioengineering, Biomaterials and Nanomedicine (CIBER-BBN), Valladolid, Spain; 7 Bioforge Lab, University of Valladolid, Edificio LUCIA, Paseo de Belen, 19, Valladolid, Spain

**Keywords:** electrospinning, silk, elastin-like-recombinamers, tissue engineering

## Abstract

In the field of tissue engineering the choice of materials is of great importance given the possibility to use biocompatible polymers produced by means of biotechnology. A large number of synthetic and natural materials have been used to this purpose and processed into scaffolds using Electrospinning technique. Among materials that could be used for the fabrication of scaffold and degradable membranes, natural polymers such as collagen, elastin or fibroin offer the possibility to design structures strictly similar to the extracellular matrix (ECM). Biotechnology and genetic engineering made possible the advent of a new class of biopolymers called protein-based polymers. One example is represented by the silk-elastin-proteins that combine the elasticity and resilience of elastin with the high tensile strength of silk-fibroin and display engineered bioactive sequences. In this work, we use electrospinning technique to produce a fibrous scaffold made of the co-recombinamer Silk-ELR. Obtained fibres have been characterized from the morphological point of view. Homogeneity and morphology have been explored using Scanning Electron Microscopy. A thorough study regarding the influence of Voltage, flow rate and distance have been carried out to determine the appropriate parameters to obtain the fibrous mats without defects and with a good distribution of diameters. Cytocompatibility has also been *in vitro* tested. For the first time we use the co-recombinamer Silk-ELR for the fabrication of a 2.5 angioplasty balloon coating. This structure could be useful as a coated scaffold for the regeneration of intima layer of vessels.

## Introduction

One of the most important aspects in the manufacture of scaffolds for tissue engineering is to be able to have access to materials mimicking the extracellular matrix (ECM). Recently, it also drew attention the use of naturally derived materials, which couple non-toxicity to other important properties [1]. As we previously demonstrated, the elastin-like-recombinamers can be used for the fabrication of biodegradable scaffolds for tissue regeneration [2]. The scaffold should be structurally and morphologically similar to the extracellular matrix (ECM). Therefore, important tasks are the cell–material interaction, sensitivity to proteases and the release of cytokines. The ECM has a fibril composition and a viscous character and such structure significantly influence the cell–biomaterial interaction [1].

With the improvement of recombinant technology and the aid of recombinant DNA it is possible to create polypeptide molecules that possess properties of two or more peptides. This new class of protein-based materials, called recombinant protein-based polymers (rPBPs) with precisely controlled polypeptide sequences, mimics the properties of their natural counterparts but can also display functions and properties not found in nature [3]. Silk, popularly known in the textile industry is a biomaterial useful for tissue applications and regenerative medicine. Silk is a fibrous protein, mostly composed by hydrophobic domains consisting of short-side chain aminoacids, synthesized in specialized epithelial cells of a great number of organisms such as spiders, butterflies and several worms of the order of Lepidoptera [4]. Biocompatible and biodegradable silk-fibroin have been widely used as base material for the fabrication of scaffolds [5].

As most of natural materials silk can be processed into foams, films meshes and fibres [5]. Silk proteins are biocompatible, biodegradable and mechanically superior thus offering a wide range of properties that are amenable to aqueous or organic solvent processing and can be chemically modified to suit a wide range of biomedical applications [6]. Due to its characteristics, silk fibroin offers versatility in matrix scaffold design for a number of tissue engineering needs in which mechanical performance and biological interactions are major factors for success, including bone, ligaments, tendons, blood vessels and cartilage [5].

By combining the peculiar characteristics of silk and elastin, is possible to obtain a copolymer that combines the properties of both proteins. Generally, Silk-ELR copolymers are composed of silk-like and elastin-like repeating units with aminoacid sequences—GAGAGS (G: Glicyne; A: Alanine; S: Serine) and V_1_PG_1_V_2_G_2_ (V: Valine; P: Proline), respectively [3, 7]. The elastin-like unit displays an highly flexible conformation [7], while the silk unit spontaneously self-assembles into packed antiparallel β-structures stabilized by hydrogen-bonding [8].

Several Silk-ELR combinations with different size and silk to elastin ratio have been used and are reported in the literature. Depending on their specifically sequence and composition, they are able to display a sol–gel transition at body temperature. This peculiar transition is a reversible process that stops as the temperature is lowered. Silk-ELR co-recombinamer has a dual gelling behaviour. The first gelation process is due to the reversible transition temperature of the—VPGIG block. Silk block causes the maturation of the gel due to the association and stabilization of β-structures [1].

As previously demonstrated, electrospinning technique allows the fabrication of biofunctionalized elastin-like-polypetide nanofibres using different process parameters [2, 9]. One of the major advantages associated with the use of electrospinning is the enormous versatility and rapidity of the process that can allow the fabrication of a porous matrix structure. Fibres electrospun from aqueous solutions of *Bombyx mori* fibroin and PEO were used as a scaffold for human aortic endothelial cells and human coronary artery smooth muscle cells [10]. Electrospun fibre mats of Silk-ELR copolymers can be interesting candidates for applications in tissue regeneration and wound dressing as the design of these particular molecules combines the tensile strength of silk and the resilience of elastin in a single molecule [3].

In this work, fibres of Silk-ELR co-recombinamer were obtained. With a thorough study of process parameters we obtain bioactive scaffolds that were preliminary *in vitro* tested for the adhesion and efficacy. We produced and characterized a nanofibrous scaffold for the regeneration of intima layer of vessels. Moreover, we realized a new coated angioplasty catheter that could act as a new bioresorbable scaffold.

## Materials and methods

**Figure 1:**
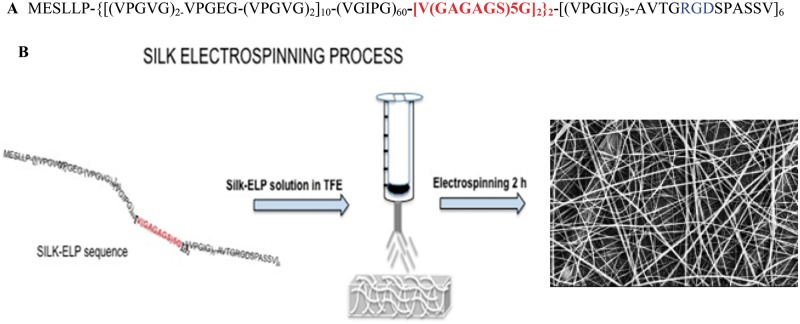
Co-recombinamer Silk-ELR primary structure (**A**). The highlighted blue part is the SILK portion, while the red one is the RGD bioactive sequence. Scheme of Silk-ELR co-recombinamer electrospinning and related fibre mats obtained (**B**)

Silk-ELR recombinamers were kindly provided by Technical Proteins NanoBiotechnology S.L. Fig. 1A shows the polypeptide composition. The cell binding domain—RGD is flanked by VPGVG/VPGEG pentapeptide block repeats that confers elastic properties while the silk block—GAGAGS give stability and resilience to the structure [7].

2,2,2-Trifluoroethanol (CAS nr. 75-89-8, purity ≥99%) was used to dissolve the silk-polymer. TFE easily dissolves both the elastin block and the silk one, leading to a clear solution. In a previous work, we used the same solvent to produce clear elastin-like polypeptide solutions, demonstrating the influence of the fluoroalcohol in modifying the molecule arrangement and influencing the aggregate size [2]. Silk-ELR co-recombinamer phase transition temperature causes the rapid auto-assembling of the silk block; so, after the addition of TFE, solutions were promptly electrospun to avoid the gelation. A schematic panel of the electrospinning process is reported in Fig. 1B.

The aim of this work is the fabrication of a porous scaffold made of a silk-elastin like recombinamer polymer that could be useful as scaffold for tissue regeneration. Preliminary *in vitro* assays were conducted with human umbilical vein endothelial cells (Invitrogen, LIFE-TECHNOLOGIES, Italy) to evaluate the adhesion and proliferation of cells on top of ELR scaffolds. Viability of cells on top of nanofibrous scaffolds was measured using Sytox Green and Phalloidin staining (Sigma-Aldrich, Italy).

## Preparation of silk-ELR fibrous mats

Silk-ELR polymer (MW 12 KDa) was suspended in 2,2,2-trifluoroethanol at concentration of 0.10%(w/v). The solutions were maintained under stirring for 5 min at room temperature and then were promptly electrospun, to avoid the gelation process. We made a thorough study to evaluate the best setting of process parameters. Nanofibres from Silk-ELR co-recombinamers are formed using voltage values between 13 and 20 kV and feed rates between 0.2 and 0.5 ml/h. All the Electrospinning experiments were conducted with Nanofibre NF-500 Electrospinning Machine (MECC-LTD, Japan). Polymer solutions were placed in plastic syringes of 6 ml (Norm-Ject Luer Lock) and fitted by an 18 × 15 mm needle. Fibres were collected on a flat aluminium collector with varying tip-to-collector distance from 15 to 17 cm. All the flat matrices were left under hood for 24 h, prior to Scanning Electron Microscopy analysis. All surfaces covered with Silk-ELR recombinamers were observed using Scanning Electron Microscopy (FESEM-ULTRAPLUS, Zeiss) in a low vacuum mode. Samples were sputter coated with 10 nm Au (CRESSINGTON 208 HR, High resolution sputter coater) and all the images were collected at 10 kV. SEM images were used to determine fibre diameter and the surface homogeneity. Fibre diameters were determined by counting 50 independent fibres using ImageJ software.

We realized a Silk-ELR coated angioplasty stent that could be useful for tissue regeneration we were able to realize the Silk-ELR angioplasty stents coatings with a well-attached layer of material by using the rotating mandrel of the electrospinning machine. We performed thickness measurements by using Scanning Electron Microscopy.

## Cell culture assay

Human umbilical vein endothelial cells—HUVECs (Invitrogen, LIFE-TECHNOLOGIES, Italy) were maintained with M200 medium (Gibco^®^, LIFE-TECHNOLOGIES, Italy) and supplemented with low serum supplement kit (LGS kit-Gibco^®^, LIFE-TECHNOLOGIES, Italy) containing 2% of foetal bovine serum (FBS), basic fibroblast growth factor (3 ng/ml), human epidermal growth factor (10 ng/ml), hydrocortisone (1 mg(ml), heparin (10 mg/ml) and Penicillin/Streptomycin (100 U ml^−1^/100 µg ml^−1^), at 37°C in a humidified atmosphere comprising 5% CO_2_ and 95% air.

All the Silk-ELR surfaces used for *in vitro* assays were previously sterilized under hood for 1 h using UV light. The medium was changed every day (for 4, 8 and 24 h) during experiments. Cell passages 5–8 were used in all cells experiments. HUVECs were seeded at a density of 10^4^ cells per scaffold on top of dried Silk-ELR surfaces placed on 24-well culture plates and on top of Gelatin (0.1%) coated dishes (control samples). Samples were divided into two sets (three samples for every time point). They were treated in the same way and cells were seeded at the same concentration (10^4^ cells).

One set of sample was used for testing cell growth. After 4, 8 and 24 h cells were removed through Trypsin treatments. We made four different Trypsinization treatments to be sure that every cell was detached from the scaffold. Therefore, ELR and Silk-ELR scaffolds were washed twice with Phospate-buffer solution (PBS). The number of cells adhered during the time of experiment was counted. The other set of sample was used to assess vitality and adhesion of cells to our scaffolds. Cells were fixed with 4% Paraformaldehyde (Sigma-Aldrich, Italy). Sytox Green (Invitrogen, LIFE-TECHNOLOGIES, Italy) and Phalloidin were used to visualize cell nuclei and cytoskeleton. Morphology and spreading of stained cells was collected using Confocal Transmission Microscopy (CLSM LEICA, TCS SP5). All the experiments were done in triplicate for all substrates.

## Catheters coating

Angioplasty catheters were provided by Conic Vascular (Switzerland). Total length of the catheters was 140 cm with a guide of 24.5 cm, ending with the inflating balloon. The balloons were totally biocompatible and compliant for the patients, due to the material used for the fabrication of the catheters (polyamide) and their related technical characteristics.

Catheters with a central balloon length of 2.5 mm were used for the realization of the coated scaffold. Silk-ELR solutions were directly electrospun on top of the balloon with the help of a rotating mandrel. We electrospun Silk-ELR solutions for 2 h to obtain a thicker matrix and a well-defined covered structure. The obtained Silk-ELR covered balloons were analysed by Scanning Electron Microscopy to investigate the thickness and the fibre morphology.

## Statistical analysis

Data presented are expressed as mean ± standard deviation of mean (SD). Differences between groups were assessed using ANOVA test. Comparisons between groups were performed by Bonferroni post-hoc test. In particular, statistical significance between groups was done making multiple comparisons using Bonferroni post-hoc test and it was set at *P* < 0.001.

## Results and discussion

Silk-ELR recombinamer was processed into fibres using electrospinning technique. Solutions in TFE were promptly electrospun using different process parameters. As we previously demonstrated fluoroalcohols can be used to dissolve elastin-like-recombinamers. The presence of high percentage of 2,2,2-trifluoroethanol affects the molecular size aggregate in different way, depending on the aminoacid composition [2]. In this case, the transition to a gel-like structure is influenced by the Silk block, thus inducing the faster gelation of the polymer. Electrospinning process was firstly carried out to set the best solution concentration, allowing the formation of the Taylor cone. We made a thorough study of the process parameters to obtain fibres without beads and defects. By changing tip-to-collector distance, flow rates and Voltage values, we obtain a huge variety of structures with an average size in the range of nanometers.

**Figure 2:**
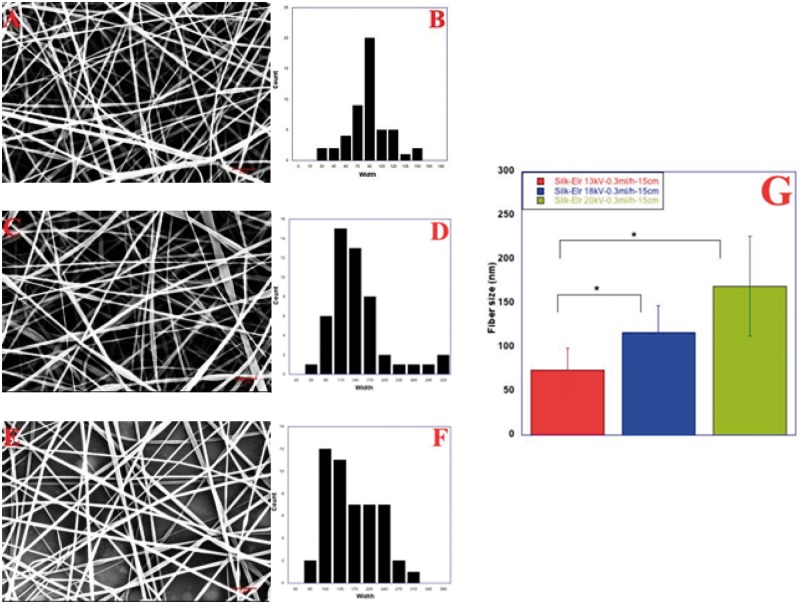
Co-recombinamer Silk-ELR fibres obtained by changing voltage values from 13 to 20 kV (**A**, **C** and **E**) and related distribution diameter histograms (**B**, **D** and **F**). Tip-to-collector distance and flow rate were kept respectively at 15 cm and 0.3 ml/h. Histogram of fibre size as a function of changing tip-to-collector distance (**G**). *P-*values < 0.001

Figure 2 shows Scanning Electron Microscopy images of Silk-ELR recombinamer obtained by changing voltage values, from 13 to 20 kV. According to the literature, increasing the voltage values allows the increase in fibre size. In particular, the lower voltage value (13 kV, Fig. 2A), together with a flow rate of 0.3 ml/h favour the formation of a good solution jet. The evaporation rate of the solvent is faster but consonant with the other parameters. Fibres appear with a flat morphology and the mean size is 70 ± 50 nm. Tip-to-collector distance of 15 cm does not influence negatively the evaporation rate. The increase in the voltage to 18 kV seems to have a negative effect on fibre morphology. Beads are more present within the sample and the fibre size is 115.5 ± 30 nm. At 20 kV, fibres appear more consistent in their morphology and less flat. As it can be seen from the picture, voltage seems to affect the amount of material deposited on top of the collector (Fig. 2E). In this case, fibre size is around 156.7 ± 70 nm. Related data of size distribution are in accordance with our experimental results. In particular, as it can be seen from Fig. 2B, D and F the better size distribution of diameter is show for first sample (Picture A, 13 kV–0.3 ml/h–15 cm).

So, as the voltage seems to have little effect on fibres morphology, we changed the feed rate and maintaining unchanged voltage values and distances.

**Figure 3:**
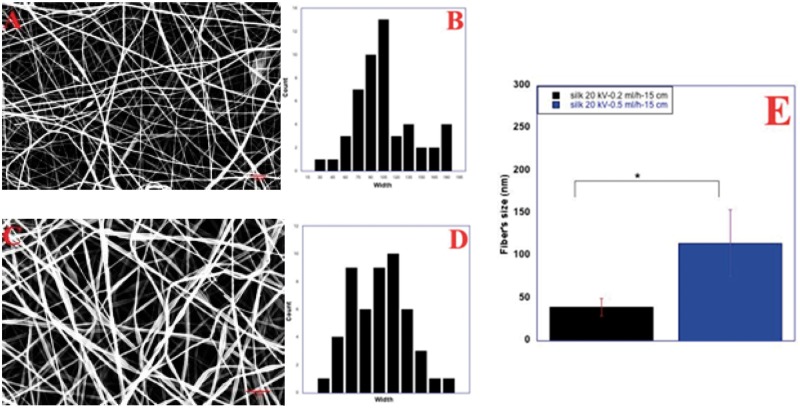
Co-recombinamer Silk-ELR fibres obtained by varying feed rate from 0.2 to 0.5 ml/h (**A** and **C**) and related distribution diameter histograms (**B** and **D**). Voltage and distance were kept respectively at 20 kV and 15 cm Histogram of fibre size as a function of changing flow rate (**E**). Silk-ELR fibres obtained increasing *P*-values < 0.001

**Figure 4:**
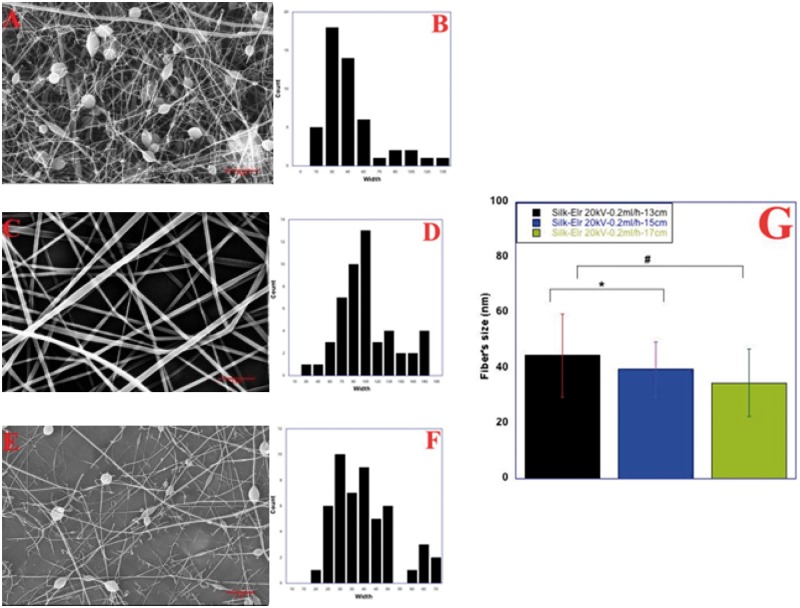
Co-recombinamer Silk-ELR fibres obtained by varying tip-to-collector distance from 13 to 17 cm (**A**, **C** and **E**) and related distribution diameter histograms (**B**, **D** and **F**). Voltage and feed rates were kept respectively at 20 kV and 0.2 ml/h. Histogram of fibre size as a function of changing tip-to-collector distance (**G**). *P-*values < 0.001

We study the effect on fibre diameter and morphology starting from 0.2 ml/h (Fig. 3A). Scanning Electron microscopy images shows fibres with a flat morphology and, in some areas, attached to each other and with a worm like morphology. The amount of material on top of the collector is consistent even though some defects are present within the sample. Fibre size is around 39.6 ± 3 nm. At 0.5 ml/h, the amount of material ejected from the nozzle is higher, causing a higher deposition of fibres on top of the collector. As expected, fibre diameter increased to 114.8 ± 39.2 nm (Fig. 3E). For better understanding the effect of all the process parameters and optimize the fibre morphology, we changed the tip-to-collector distance, from 13 to 17 cm, while keeping constant flow rates and Voltage values (20 kV and 0.2 ml/h). Fibres obtained are morphologically different and have a different average diameter that varies from 40.2 ± 15.0 to 34.7 ± 12.20 nm (*P* < 0.001). In particular, at 13 cm with 20 kV and 0.2 ml/h fibres appear broken and fool of defects. They are thinner and only few whole fibres are present (Fig. 4A). Distribution of diameters is polarized towards small values due to the thinnest size of fibres (Fig. 4B). Thinnest fibres could be the result of an increasing dielectric constant of Silk-ELR solutions.

By increasing the distance to 15 cm together with feed rate of 0.2 ml/h, fibres appear more consistent but the amount of material deposited on top of the collector seems to be lower. Unless the presence of some defects within the sample, fibres are with a flat morphology (Fig. 4C).

This behaviour should be related to the lower amount of solution ejected from the nozzle. Conversely, at 17 cm, with feed rate of 0.2 ml/h the distance is higher and the amount of material that is deposited on top of the collector is the lowest. Moreover, beads and broken fibres are present within the sample (Fig. 4E). Distribution of diameters is in accordance with our experimental results (Fig. 4D and F).

Electrospinning of ELR solutions in TFE is affected both by the chemical characteristics of the fluorinated solvent together with the peculiar properties of the polypeptides. The ELR block conversely gives elasticity to the polypeptide [7]. As we reported in a previous work, electrospinning of Elastin-like-recombinamers in Trifluoroethanol solutions can give rise to a huge variety of aggregates that increase with the increasing temperature [2].

Silk block is known to be the stable part that gives resilience and stability to the whole molecule and reported to be affected by the fluorinated alcohols, such as Trifluoroethanol [1]. Thanks to the presence of a different aminoacid sequence silk material is able to self-assemble thus favouring the stabilization of β Thanks to the presence of a different behaviour mainly affects the morphological characteristics of fibres, as it can be seen from our results.

Therefore, the best set of process parameters seems to be related to lowest values of Voltage, feed rate and distance. So, the sample with process parameters of 13 kV- 0.3 ml/h and 15 cm was used for our experiments.

## *In vitro* adhesion and viability

Biocompatibility and topographical characteristics affect cell behaviour. In particular, cell fate is known to be influenced by the recognition of specifically bioactive sequences that have to be present within the biomaterial. The Silk-ELR co-recombinamer fibre matrices used matches together the biocompatibility of the material and the presence of a well-known bioactive sequence, such as RGD.

**Figure 5:**
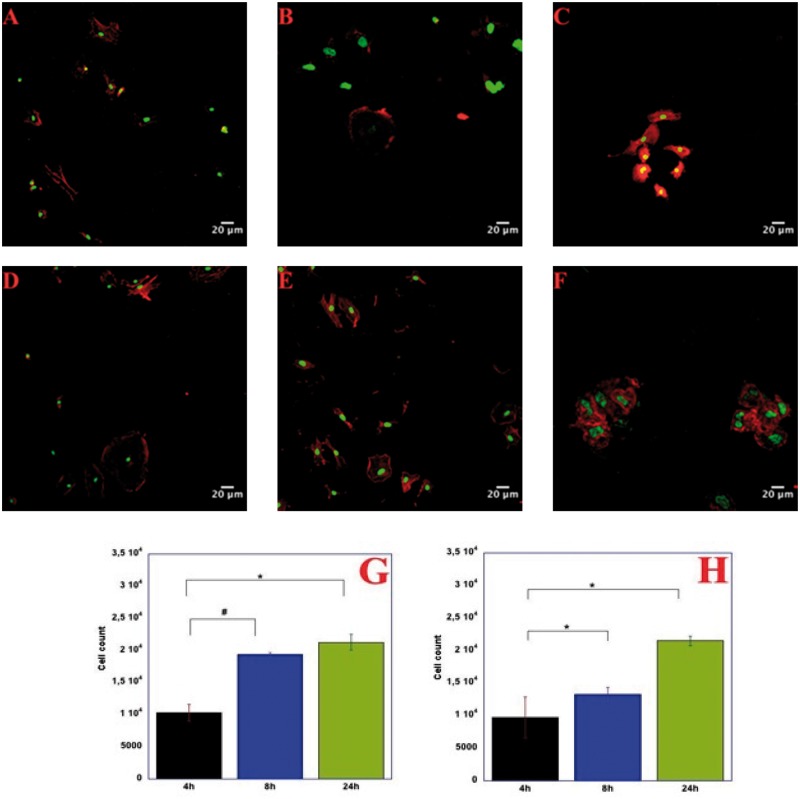
Confocal Microscopy images of HUVECs on gelatin coated dishes (**A**, **B** and **C**) and the respective histogram with adhesion of cells as a function of seeding time (**G**). HUVECs on Silk-ELRs co-recombinamer scaffolds (**D**, **E** and **F**) and the related histogram with adhesion of cells as a function of seeding time (**H**). Scale bar 20 µm. *P*–values < 0.001

Figure 5 reported the adhesion and the early proliferation of HUVECs on top Gelatin (0.1%) coated controls (A, B and C) versus Silk-ELR scaffolds (D, E and F). at 4 h the adhesion of cells is almost similar for both the two types of substrates (Fig. 5G and H).

Conversely, at 8 h there is a huge difference of adhesion rate between the gelatin coated controls and the Silk-ELR scaffolds. In fact, for the control there is an increasing adhesion of cells to the substrate due to the stable environment of gelatin coated dishes and the familiarity of cells for this type of substrate (Fig. 5B and G). On the contrary, at 8 h, there is no evident difference in the adhesion for the Silk-ELR scaffolds. This behaviour could be attributed to an initial and crucial phase of acclimatization of cells on top of this substrate (Fig. 5E and H).

An increase in adhesion of cells that shifts from 1 × 10^4^ to 2 × 10^4^ is evident for the control samples (Fig. 5G), while for the Silk-ELR scaffolds the adhesion rate increases a little and settles to 1.1 × 10^4^ (Fig. 5H), but the spreading of cells is more evident (Fig. 5E). At 24 h, there is an evident increase in the adhesion rate of cells on top of the scaffolds.

The HUVEC behaviour demonstrated that Silk-ELR scaffolds are totally biocompatible and safe. Moreover, the presence of specific recognition sequences within the polypeptide allows the increasing adhesion of cells to the scaffolds. An initial proliferation phase is detectable at 24 h, as shown in Fig. 5H.

## Silk-ELR coated balloons

**Figure 6:**
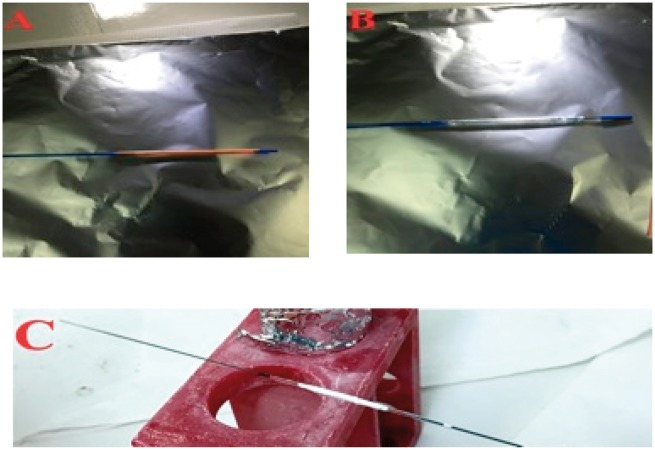
Images of angioplasty catheters provided by Conic Vascular and used for the fabrication of Silk-ELR matrices. Balloon with its coverage (**A**) and without the coverage (**B**). Fibre matrix obtained by electrospinning Silk-ELR solutions for 2 h (**C**)

Catheters provided by Conic Vascular are depicted in Fig. 6. In particular, Fig. 6 shows the angioplasty balloons with its coverage (A) and without it (B). The central part is delimited by two silver lines that are present at each extremity of the balloon (Fig. 6B).

Electrospinning of Silk-ELR solutions for 2 h allows the obtaining of a well-adhered scaffold around the central part of the catheter. The result of the process is shown in Fig. 6C. As it can be seen from the picture, Silk-ELR fibre matrix is visible around the central portion of the balloon. There are no macro defects visible on the sample.

**Figure 7:**
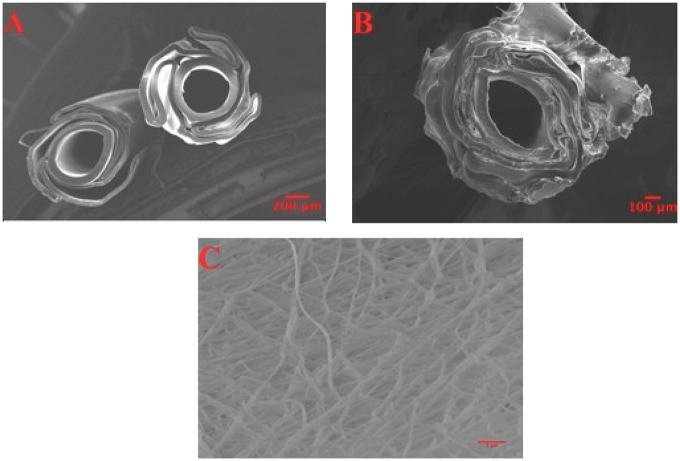
Scanning Electron Microscopy angioplasty catheters structure (**A** and **B**). Picture A shows the internal part of the balloon without the Silk-ELR fibre matrix. Picture B shows the structure of the balloons with the adhered Silk-ELR matrix around it. Scanning Electron Microscopy was used for the evaluation of fibre morphology (**C**). Picture **D** shows Scanning Electron Microscopy measurements of layer thickness

Scanning Electron Microscopy images of the Silk-ELR scaffolds were acquired to study the morphological characteristics of fibres attached to the balloons. Figure 7 shows control balloons (A) versus Silk-ELR covered stents (B). From the images acquired is possible to recognize the typical internal balloon structure that is characterized by a lamellar body organized around a central part (A). Electrospinning of Silk-ELR solutions around the balloon let the insertion of the fibrous material into the internal lamellar-like body of the stent (B). Texture of the fibrous matrix seems to be disordered but well attached to the stent.

Silk-ELR fibres around the balloon form an interconnected matrix (C). Morphology of fibres around the angioplasty balloon is in accordance with our previous results. Fibres are thinnest and there are no beads or defects within the sample (C).

Moreover, to make a thorough study on the thickness of fibre’s layer, Scanning Electron Microscopy was used. In fact, Silk-ELR angioplasty scaffold obtained seems to have the same thickness in different points of our samples. Using the Scanning Electron Microscopy measurement tools we were able to make measurements of the thickness of stents in twenty points. Our result shows that the thickness of the coated stents is homogeneous and well attached to the angioplasty balloon. In particular, thickness was 20.14 ± 2.1 µm.

## Conclusions

In this work, we processed a new biotechnological material made by combining a Silk block with an Elastin derived sequence conceived as scaffold for intima regeneration producing fibrous balloon coating. In particular, such new material has been processed into fibres using Electrospinning technique. As we show in our work, fibres obtained are good in fibre size and morphology for all the samples with respect to the proposed application. Moreover, distribution of diameter is in accordance with our experimental results. In particular, for our *in vitro* experiments and for the fabrication of coated stents we used samples with lower values of voltage and feed rates (13 kV, 0.3 ml/h, 15 cm).

The selected process parameters allow good biocompatibility and good adhesion and proliferation of cells, as our *in vitro* adhesion test demonstrated. Adhesion of HUVECs is stable for Silk-ELR scaffolds: cells maintained in culture for 24 h are well adhered to the substrate and the early proliferation phase of cells is higher at 24 h respect to the control samples.

Therefore, we demonstrated Electrospinning technique can be used for the fabrication of different kinds of Elastin-like-polymer scaffolds. In particular, in this work, we obtain a coated stent that could be useful for different cardiovascular regeneration of intima layer of vessels approaches. In this case, we use our SILK-ELR material solutions for the fabrication of angioplasty stents coating. We demonstrated that produced stent coatings are uniform in thickness and the fibres are appropriate in morphology and size for the specific application.

## Funding

This work was supported by funds provided by “THE GRAIL” (Tissue in Host Engineering Guided Regeneration of Arterial Intima Layer) project. The project is funded by the European Union’s ‘Seventh Framework’ Programme for research, technological development and demonstration under Grant Agreement no. HEALTH.2011.1.4-2-278557. Authors would also acknowledge the following institutions: European Commission (NMP-2014-646075, MSCA-ITN-2014-642687), MINECO of the Spanish Government (PCIN-2015-010, MAT2015-68901-R, MAT2016-78903-R), Junta de Castilla y León (VA015U16) and Centro en Red de Medicina Regenerativa y Terapia Celular de Castilla y León.

*Conflict of interest statement.* None declared.
